# In response to partial plant shading, the lack of phytochrome A does not directly induce leaf senescence but alters the fine-tuning of chlorophyll biosynthesis

**DOI:** 10.1093/jxb/eru060

**Published:** 2014-03-06

**Authors:** Bastiaan Brouwer, Per Gardeström, Olivier Keech

**Affiliations:** Umeå Plant Science Centre, Department of Plant Physiology, Umeå University, S-90187 Umeå, Sweden

**Keywords:** *Arabidopsis*, chlorophyll, far-red light, phytochrome, senescence, shade.

## Abstract

Phytochrome is thought to control the induction of leaf senescence directly, however, the signalling and molecular mechanisms remain unclear. In the present study, an ecophysiological approach was used to establish a functional connection between phytochrome signalling and the physiological processes underlying the induction of leaf senescence in response to shade. With shade it is important to distinguish between complete and partial shading, during which either the whole or only a part of the plant is shaded, respectively. It is first shown here that, while PHYB is required to maintain chlorophyll content in a completely shaded plant, only PHYA is involved in maintaining the leaf chlorophyll content in response to partial plant shading. Second, it is shown that leaf yellowing associated with strong partial shading in *phyA*-mutant plants actually correlates to a decreased biosynthesis of chlorophyll rather than to an increase of its degradation. Third, it is shown that the physiological impact of this decreased biosynthesis of chlorophyll in strongly shaded *phyA*-mutant leaves is accompanied by a decreased capacity to adjust the Light Compensation Point. However, the increased leaf yellowing in *phyA*-mutant plants is not accompanied by an increase of senescence-specific molecular markers, which argues against a direct role of PHYA in inducing leaf senescence in response to partial shade. In conclusion, it is proposed that PHYA, but not PHYB, is essential for fine-tuning the chlorophyll biosynthetic pathway in response to partial shading. In turn, this mechanism allows the shaded leaf to adjust its photosynthetic machinery to very low irradiances, thus maintaining a positive carbon balance and repressing the induction of leaf senescence, which can occur under prolonged periods of shade.

## Summary

In partially shaded plants, phytochrome A contributes to the adjustment of photosynthesis in shaded leaves but is not directly involved in the induction of leaf senescence

## Introduction

For most plants, survival and reproductive capacity depend on an ability to optimize photosynthetic yield and mobilize resources efficiently. Accordingly, throughout evolutionary history plants have developed adaptive strategies to cope with a wide variety of stresses. One of these adaptive strategies is leaf senescence. This genetically controlled process (Yoshida, 1962) is characterized by leaf yellowing, which results from the active degradation of chlorophyll (Pružinská *et al.*, 2005; Schelbert *et al.*, 2009), proteins (Martínez *et al.*, 2008), and nucleic acids (Buchanan-Wollaston *et al.*, 2003). The senescence-associated degradation contributes strongly to the remobilization of growth-limiting nutrients such as nitrogen, phosphorus, and sulphur from senescing organs towards other parts of the plant (Snapp and Lynch, 1996; Masclaux-Daubresse *et al.*, 2008). Besides ageing, leaf senescence can be induced and accelerated by a variety of biotic and abiotic stresses (Smart, 1994), including shade and darkness (Biswal and Biswal, 1984; Rousseaux *et al.*, 1996; Weaver and Amasino, 2001).

Interestingly, the capacity of shade and darkness to induce leaf senescence depends on whether the plant is completely or partially shaded or darkened (Weaver and Amasino, 2001; Keech *et al.*, 2007). Complete plant shading, which occurs when plants are completely overshadowed by an established canopy, often triggers a symptomatic shade-avoidance response that is characterized by increased petiole length, decreased leaf surface area, and delayed leaf yellowing (Hidema *et al.*, 1992; Franklin, 2008; Casal, 2012). Partial shading, in which only a part of the plant is shaded while the rest of the plant remains under growth light, often occurs in densely planted monoculture crops where the lower leaves are overshadowed from above or by neighbouring leaves in the canopy. Under such circumstances, the shaded leaves both balance the photochemical efficiencies of their photosystems and minimize respiration in order to reduce their Light Compensation Point (LCP) and maintain a positive carbon balance (Brouwer *et al.*, 2012). However, if the light intensity is too low, the leaf cannot acclimate sufficiently and leaf senescence is induced (Brouwer *et al.*, 2012).

Shade-avoidance responses are mediated through photoreceptors, in particular phytochromes (Franklin, 2008; Casal, 2012). Phytochromes constitute a family of photoreceptors whose native, red light absorbing form (Pr) is rapidly converted by red light (R) to its biologically active and far-red light (FR) absorbing form (Pfr). Active Pfr can subsequently be converted back to inactive Pr by FR or darkness (Franklin and Quail, 2010; Rausenberger *et al.*, 2010). Active Pfr is translocated to the nucleus, where it both promotes and inhibits the degradation of various transcription factors, e.g. Phytochrome Interaction Factors (PIFs) and HYpocotyl 5 (HY5), respectively. In turn, these mechanisms regulate a variety of photomorphogenic processes such as seed germination, de-etiolation, and shade avoidance (Bae and Choi, 2008; Franklin and Quail, 2010; Lau and Deng, 2010).

Evidence for the involvement of phytochromes in mediating leaf senescence has been provided by a number of studies showing that the loss of chlorophyll is inhibited by R and that this inhibition can be reversed by subsequent illumination with FR (Sugiura, 1963; De Greef *et al.*, 1971; Biswal and Biswal, 1984; Okada and Katoh, 1998). Studies on partial shading in sunflower and tobacco have shown that FR-enrichment under normal growth irradiances mildly accelerates leaf yellowing (Rousseaux *et al.*, 1996, 1997; Pons and de Jong-van Berkel, 2004). However, leaf yellowing is not accelerated when FR is enriched under low light conditions, e.g. shade (Borrás *et al.*, 2003; Brouwer *et al.*, 2012). Moreover, plants over-expressing phytochrome A (*PHYA*) or phytochrome B (*PHYB*) show delayed leaf yellowing (Rousseaux *et al.*, 1997; Thiele *et al.*, 1999), whereas phytochrome A mutant (*phyA*) plants show increased leaf yellowing in response to shade (Brouwer *et al.*, 2012). Yet, despite these lines of evidence showing that phytochromes affect shade-induced leaf yellowing, the connections between phytochrome-mediated signalling pathways and the physiological mechanisms underlying the induction of leaf senescence are still poorly understood.

In *Arabidopsis*, the phytochrome family consists of five members, namely PHYA, PHYB, PHYC, PHYD, and PHYE, of which PHYA, PHYB, and PHYD are predominant and best characterized (Aukerman *et al.*, 1997; Franklin and Quail, 2010). While these three phytochromes are known to mediate shade-avoidance responses, their respective contribution to the induction of leaf senescence in response to either complete or partial shading has not yet been established. In the present study, an ecophysiological approach was chosen in order to gain an insight into the regulatory mechanisms by which phytochrome signalling could control the induction of leaf senescence in response to light deprivation. Due to redundancy between PHYB and PHYD, our efforts were mainly focused on PHYA and PHYB. First, it is shown that while PHYB is required to maintain chlorophyll content in a completely shaded plant, only PHYA is involved in maintaining the leaf chlorophyll content in response to partial plant shading. Second, it is shown that the leaf yellowing associated with strong partial shading in *phyA*-mutant plants actually correlates to a decreased expression of genes related to the biosynthesis of chlorophyll rather than to an increase in its degradation. Third, it is shown that the physiological impact of this decreased biosynthesis of chlorophyll in strongly shaded *phyA*-mutant leaves is accompanied by a decreased capacity to adjust the LCP. Given these findings, it is proposed that PHYA is essential for fine-tuning the chlorophyll biosynthetic pathway in response to partial shading. This, in turn, allows the shaded leaf to adapt its photosynthetic machinery to very low irradiances, thus maintaining a positive carbon balance and repressing the induction of leaf senescence.

## Materials and methods

### Plant growth and light treatments

*Arabidopsis* Landsberg *erecta* (L*er*) wild-type (wt) and *phyA-201* lines have been described previously (Reed *et al.*, 1994), as have lines *phyA-302* (Yanovsky *et al.*, 2002) and *phyA-401* (Dieterle *et al.*, 2005). The other lines were in a Wassileweskija (Ws) background. The Ws wt, the phytochrome mutant alleles *phyA-5* and *phyB-10*, and the PHYD-expressing line (PHYD^+^) have been described by Aukerman *et al.* (1997) and Franklin *et al.* (2003).

Plants were grown in a controlled environment growth chamber with a short-day photoperiod (8/16h light/dark, 22/17 °C) at 75% relative humidity and 250 µmol m^–2^ s^–1^ white light at growth level. The short-day growth period served to increase both the number and size of the leaves, thus facilitating physiological analyses. At the age of 6 weeks after sowing, plants were used for shading or darkening treatments. Shading was done by covering individual leaves (selected from among leaf numbers 14 to 20), at most three per plant, with light reduction- or darkening-envelopes, which have been described extensively in Keech *et al.* (2007) and Brouwer *et al.* (2012).

Whole plant shading treatments were carried out using calibrated E-30 floraLED light cabinets (Percival, Perry, IA, USA).

Pulsed or continuous FR treatments were carried out by partially covering plants (30–45% of the total leaf surface) using dark-boxes that supported calibrated LED-arrays (MD Electronics, London, UK). The dark-boxes were constructed from cardboard and black plastic with styrofoam inserts in order to limit both light entry from below and to provide support for four plant pots (see Supplementary Fig. S3a available at *JXB* online).

All LED-arrays were calibrated before use and connected to plug-in digital timers to regulate either pulsed (3min h^–1^) or continuous light. In both cases, illumination treatments were initiated at the onset and terminated at the end of the light phase of the original photoperiod.

Light source calibration and light measurements were carried out using a Spectroradiometer (RPS900-R, International Light, Peabody, MA, USA) and the SpectrILight Analysis software (International Light, Peabody, MA, USA). Light spectra between 380 and 800nm were recorded for all light conditions (see Supplementary Fig. S2 available at *JXB* online). All light intensity values were determined between 400–700nm, while the R/FR ratios were calculated using intensities between 640–660nm for red light, and 720–740nm for far-red light. For Fig. 3a, b and for Supplementary Fig. S2c available at *JXB* online, the far-red light intensities were determined between 720–740nm and designated as FR.

Leaf treatments and sampling were carried out at midday. After treatment, samples were taken from the distal halves of leaf blades and then frozen in liquid nitrogen.

### Chlorophyll analysis

Chlorophyll was extracted using phosphate-buffered 80% acetone and analysed at 646.6, 663.6, and 750nm as described in Porra *et al.* (1989) and Brouwer *et al.* (2012) using a Lambda 18 Spectrophotometer (Perkin-Elmer, Waltham, MA, USA).

### qPCR

Total RNA was extracted from frozen samples—each consisting of the distal half of a leaf—using an E.Z.N.A.^TM^ Plant RNA Mini Kit (Omega Bio-Tek, Norcross, GA, USA) and treated with DNA-free^TM^ (Ambion, Austin, TX, USA). RNA concentrations were determined using a ND-2000 spectrophotometer (NanoDrop Technologies, Inc, Wilmington, DE, USA) and RNA quality was assessed by loading 200ng RNA on a 1% (w/v) agarose gel. Equal amounts of RNA (180ng) were transcribed using a qScript cDNA-synthesis kit (Quanta Biosciences, Gaithersburg, MD, USA). Primers used for qPCR are specified in the Supplementary data (see Supplementary Table S1 available at *JXB* online) and were produced by Cybergene AB (Stockholm, Sweden). Quantitative PCR (qPCR) reactions (10 μl) were performed in triplicate in BR-white plates using a CFX-96 Real-Time PCR Detection system (Bio-Rad, Hercules, CA, USA). Each reaction consisted of 4 μl cDNA (1/20×), 1 μl 10 μM FW/RV primer-mix and 5 μl B-R SYBR Green Supermix for IQ (Quanta Biosciences). The cycling program used consisted of an initial step at 95 °C for 5 min; 45 cycles of 10 s at 95 °C, 10 s at 59 °C, and 20 s at 72 °C; 10 s at 95 °C, and a melting curve from 65 °C to 95 °C at 0.5 °C increments and 5 s per increment. The resulting data were processed using CFX Manager 2.1 (Bio-Rad, Hercules, CA, USA) prior to statistical analysis. Gene expression was normalized using the expression of two reference genes, APT1 and TIP41, as previously used in Keech *et al.* (2010).

### Photosynthesis and respiration

The photosynthetic rate at 250 μmol m^–2^ s^–1^ (A_250_), the dark respiration (R_d_), and the photosynthetic light compensation point (LCP) were determined using a Li-Cor 6400XT infra-red gas analyser equipped with a 3×2cm leaf chamber (Li-Cor BioSciences, Lincoln, NE, USA). Net CO_2_ assimilation was measured over a range of decreasing light intensities (250, 75, 50, 25, 20, 15, 10, 5, 1, and 0 μmol m^–2^ s^–1^) using a blue–red LED actinic light source. During measurements of CO_2_ exchange, the leaf temperature, the relative humidity, and the CO_2_ concentration in the leaf chamber were set to 22 °C, 60%, and 400 ppm, respectively. Measurements were performed after the plants had received at least 2h of light. After each measurement, the leaf area was determined by delimiting the perimeter of the leaf on a sheet of paper and by weighing the paper outline of this area. The conversion weight to area was further used to calculate photosynthesis on an area basis.

### Data and statistical analysis

Data were prepared using Excel 2003 (Microsoft, Redmond, WA, USA). Graphs were drawn and statistical analyses performed using Prism 5 (GraphPad Software, La Jolla, CA, USA).

## Results

### Partially shaded plants require PHYA to maintain chlorophyll content in their shaded leaves

**Fig. 1.**
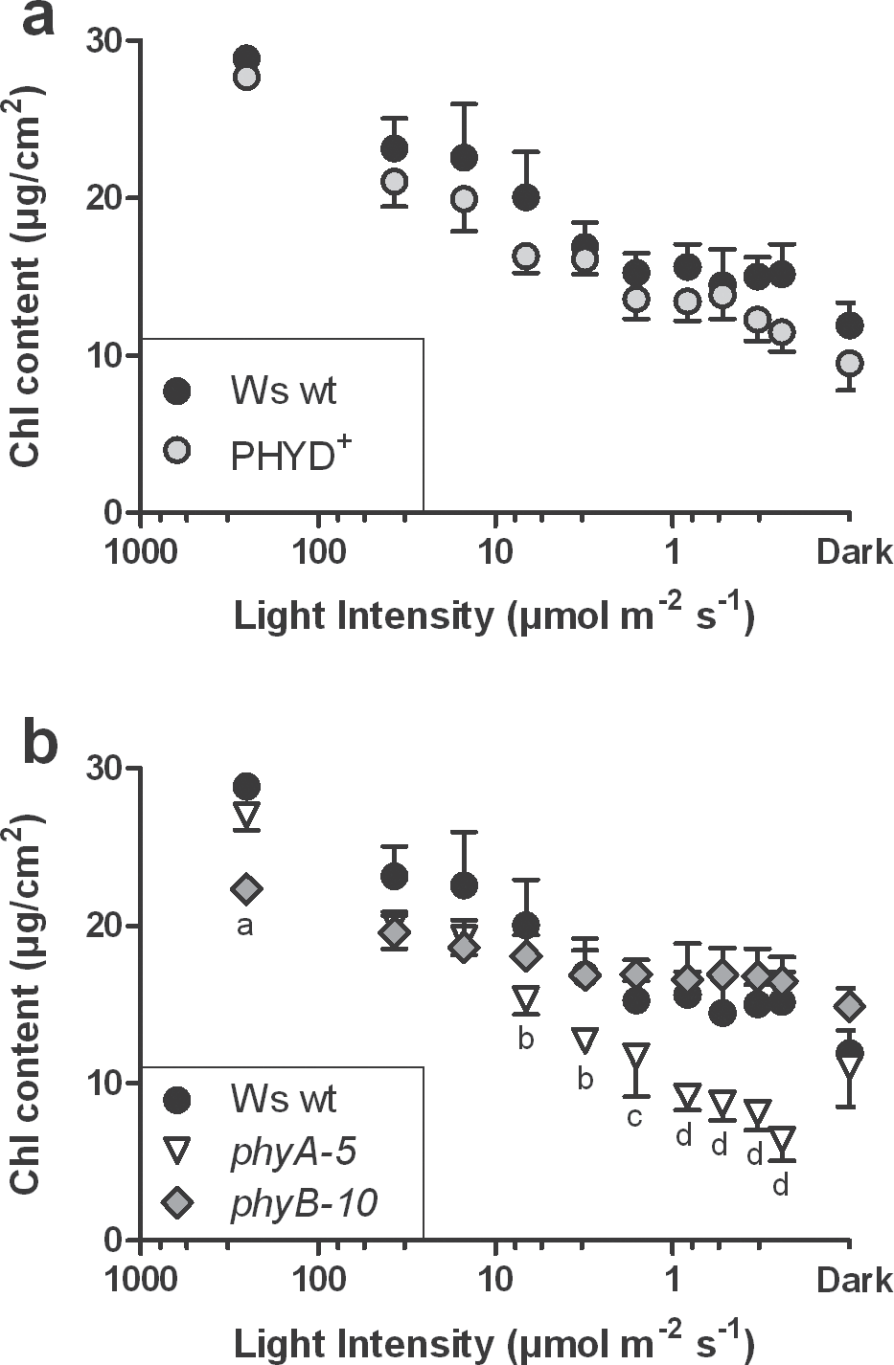
Chlorophyll content in shaded leaves of partially shaded Ws wt and phytochrome mutant plants. Leaves were either unshaded (250 μmol m^–2^ s^–1^), individually shaded, or individually darkened (Dark) for 6 d. (a) Ws wt (black circles) and PHYD-complemented PHYD^+^ (grey circles); (b) Ws wt, *phyA-5* (white triangles) and *phyB-10* (grey diamonds). Values are means ±95% CI, *n* ≥7. Notations indicate statistically significant differences (*P* <0.05) from Kruskall–Wallis with Dunn’s multiple comparison tests, between genotypes: ‘a’, wt and *phyA-5*; ‘b’, wt; ‘c’, *phyB-10*, and ‘d’, wt and *phyB-10*.

To assess which phytochrome was involved in mediating the loss of chlorophyll in response to partial plant shading, mature *Arabidopsis* wild-type (wt) and the two null-mutant plants phytochrome A (*phyA-5*) and phytochrome B (*phyB-10*) were used. These lines were chosen from a Wassileweskija (Ws) ecotype, which is a natural *phyD*-mutant (Aukerman *et al.*, 1997; Franklin *et al.*, 2003), in order to circumvent the redundancy between *PHYB* and *PHYD*. An additional *PHYD*-containing Ws-line (PHYD^+^) was also included as a control to estimate the influence of *PHYD* under partial shading. Partial shading was obtained by covering individual leaves from 6-week-old plants with envelopes made of stacked layers of water-resistant paper. As described in Brouwer *et al.* (2012), this technique can provide a range of shade from 37 down to 0.25 μmol m^–2^ s^–1^ in addition to the standard growth light intensity (250 μmol m^–2^ s^–1^) and darkness (0 μmol m^–2^ s^–1^). After 6 d, for both Ws wt and PHYD^+^ plants, the chlorophyll content of the shaded leaves had decreased, the decrease being most pronounced at the lowest light intensities (i.e. in stronger shade). However, no significant differences were observed between Ws wt and PHYD^+^ plants (Fig. 1a). Furthermore, *phyB-10* leaves contained significantly less chlorophyll than Ws wt leaves under standard growth light intensity but, interestingly, when compared with Ws wt plants, the mutation had no effect on the chlorophyll content in response to shading (Fig. 1b). By contrast, *phyA-5* leaves were indistinguishable from the Ws wt leaves, either under growth light or when darkened; instead they showed significantly lower chlorophyll content than Ws wt leaves when shaded below 10 μmol m^–2^ s^–1^ (Fig. 1b). The use of a *phyA phyB* double mutant highlighted an additive effect of the two mutations: the chlorophyll content in the double mutant leaves was lower than in Ws wt leaves both under standard growth light and shading treatments (see Supplementary Fig. S1 available at *JXB* online). These results suggest that, in partially shaded plants, PHYA is required to limit the loss of chlorophyll in shaded leaves, but PHYB and PHYD are not.

### Completely shaded mature plants require both PHYB and PHYB-activating light to maintain their chlorophyll levels

**Fig. 2.**
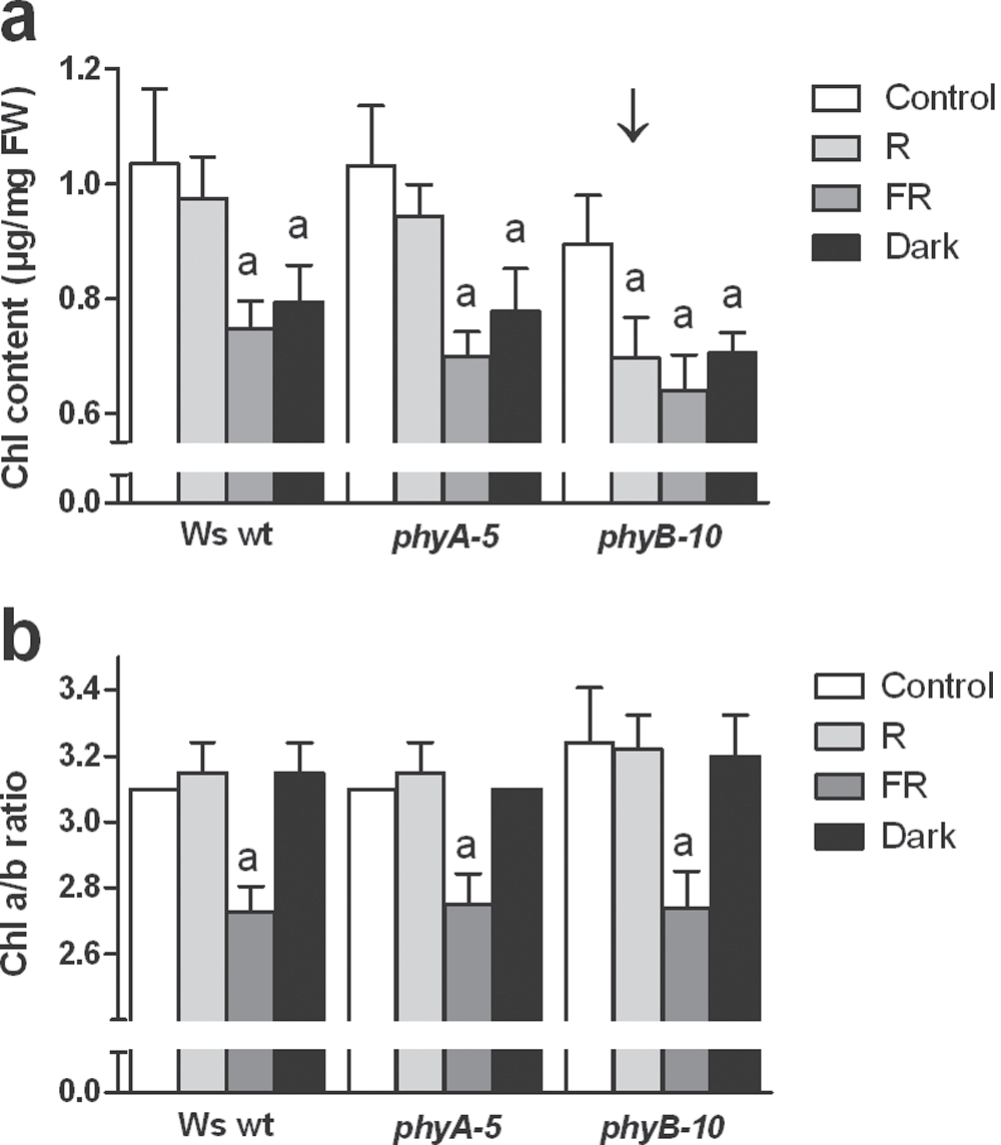
(a) Chlorophyll content, and (b) chlorophyll *a*/*b* ratio in leaves of completely shaded Ws wt, *phyA-5*, and *phyB-10* plants. Plants were either unshaded (Control; 250 μmol m^–2^ s^–1^), completely shaded at high R/FR ratio (R; 3 μmol m^–2^ s^–1^; no FR), completely shaded at low R/FR ratio (FR; 3 μmol m^–2^ s^–1^; R/FR ratio of 0.0007) or darkened (Dark) for 6 d. Values are means ±95% CI, *n* ≥4. The arrow points to the altered response. Notations indicate statistically significant (*P* <0.001) decreases from two-way ANOVA with Bonferroni post tests.

An earlier study showed that darkening a couple of leaves for 6 d triggered an accelerated senescence in these leaves, while leaves from a plant entirely darkened over the same period of time exhibited a typical shade-avoidance response with leaf hyponasty and petiole elongation (Keech *et al.*, 2007). Therefore, it was explored whether different phytochromes were involved in the regulation of the chlorophyll content under partial and complete shading of the plant. Six-week-old plants, grown under short days at growth light (250 μmol m^–2^ s^–1^; Control), were transferred to different shade conditions: low red light with a high R/FR ratio (3 μmol m^–2^ s^–1^; R), low red light with a low R/FR ratio (3 μmol m^–2^ s^–1^; FR), and darkness (0 μmol m^–2^ s^–1^; Dark) (see Supplementary Fig. S2 available at *JXB* online). After 6 d, both the Ws wt and phytochrome mutant plants had a reduced chlorophyll content under FR and Dark treatments (Fig. 2a) when compared with the Control. Interestingly, the decrease of irradiance without modification of the R/FR ratio (i.e. R) only affected the chlorophyll content in the *phyB*-mutant (Fig. 2a, arrowed); no significant decrease in the chlorophyll content was observed in the Ws wt or *phyA*-mutant plants. By contrast, under all light conditions, the chlorophyll a/b ratio behaved similarly among all genotypes: the ratio significantly decreased only under low irradiance enriched with far-red light (FR; Fig. 2b). These data show that, in contrast to leaves under partial shading, leaves from completely shaded mature plants require PHYB and PHYB-activating light to delay leaf yellowing when strongly shaded (i.e. 3 μmol m^–2^ s^–1^). In addition, the fact that the decrease of chlorophyll in *phyB*-mutant plants under R conditions does not correlate with a decrease in the *a*/*b* ratio shows that both chlorophyll *a* and *b* are equally affected by the drastic drop in irradiance.

### *PHYA* limits shade-induced loss of chlorophyll via the FR-HIR

**Fig. 3.**
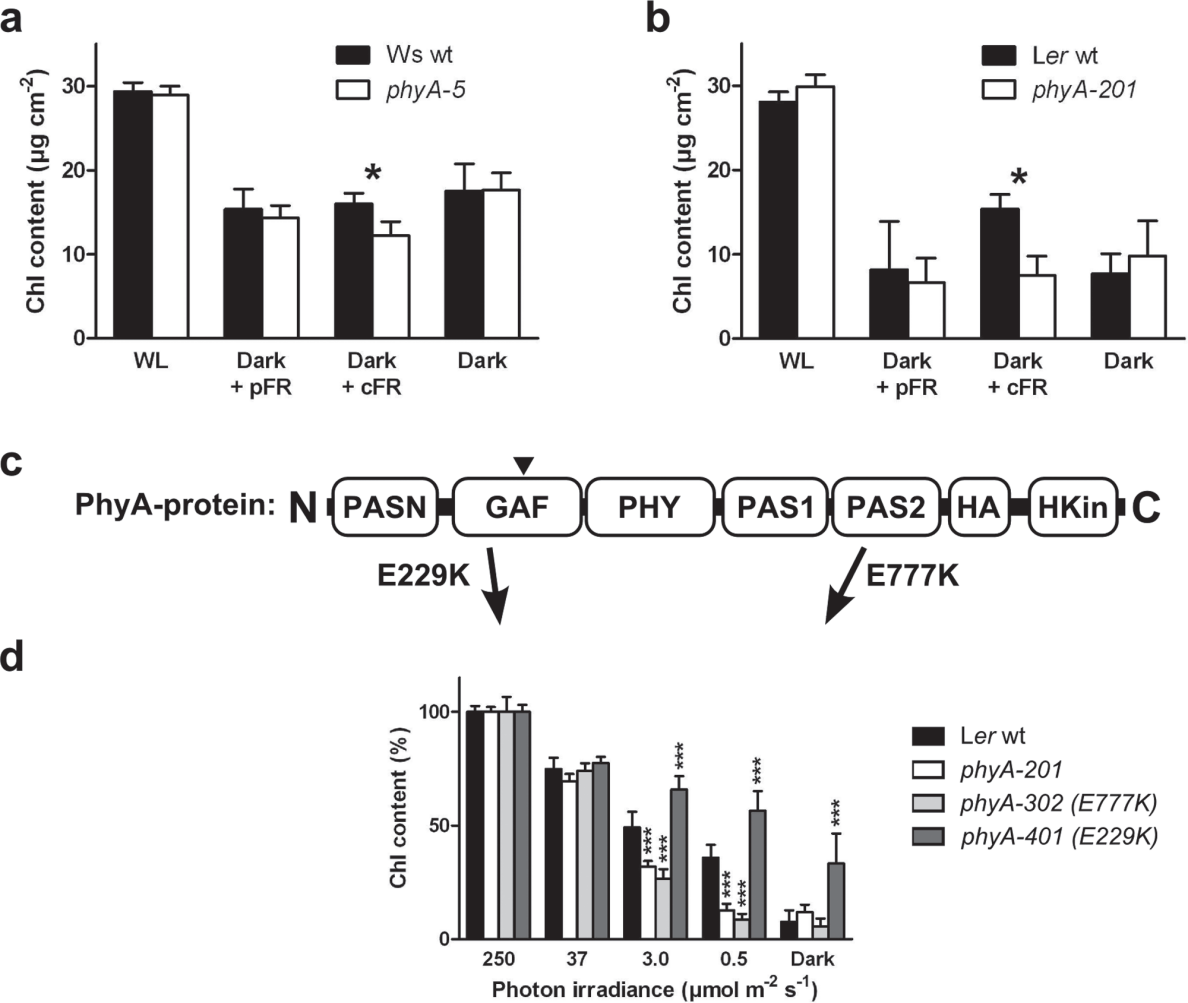
Signalling and stability of PHYA-protein. (a) Chlorophyll content in shaded leaves of Ws wt (black) and *phyA-5* plants (white) that had been partially darkened for 6 d, either with (pFR and cFR) or without (Dark) an additional FR treatment during daylight hours. Non-darkened leaves were used as a white-light control (WL). The FR treatment consisted of either hourly high-fluence pulses (pFR; 3min, 260 μmol m^–2^ s^–1^ FR) or continuous low-fluence light (cFR; 60min, 13 μmol m^–2^ s^–1^ FR) to induce either a VLFR or a HIR, respectively. Values are means ±95% CI, *n* ≥4. Statistically significant (*P* <0.05) differences from Mann–Whitney tests are notated *. (b) As (a) but using L*er* wt (black) and phyA null-mutant *phyA*-*201* (white). (c) Representation of different domains in the phyA-protein, adapted from Müller *et al.* (2009), to highlight the missense mutations used in this study. Abbreviations: C, C-terminal domain; PASN, N-terminal PER/ARNT/SIM domain; GAF, domain identified in cGMP-regulated cyclic phosphodiesterases/adenyl cyclases/bacterial transcription factor FhlA; PHY, phytochrome domain; PAS1 and PAS2, PER/ARNT/SIM domains; HA, His Kinase acceptor domain; HKin, ATP-binding His kinase-like domain; N, N-terminal domain. The chromophore-binding site within the PHYA protein is indicated by ▼. (d) Chlorophyll content in shaded leaves of partially shaded L*er* wt (black), *phyA-201* (white) and missense mutants *phyA*-302 (light grey) and *phyA-401* (dark grey) plants. Leaves were either unshaded (250 μmol m^–2^ s^–1^), individually shaded or individually darkened (Dark) for 6 d. Values are means ±95% CI, *n*=6. Statistically significant (*P* <0.001) differences from a one-way Anova with Bonferroni’s multiple comparison test compared to L*er* wt are notated: ***.

To confirm that the enhanced loss of chlorophyll in response to partial shading was due to an altered PHYA-dependent signalling and not to another developmental effect, the fact that PHYA requires FR for its translocation to the nucleus and the subsequent transduction of its signal was exploited (Franklin and Quail, 2010; Rausenberger *et al.*, 2011). In addition, by modulating the frequency and intensity of the FR dosage, two different modes of PHYA-mediated signal-transduction can be distinguished: the very low fluence response (VLFR) and the far-red high irradiance response (FR-HIR) (Casal *et al.*, 2000). While the VLFR responds to light fluences that are experienced discontinuously, the FR-HIR requires continuous irradiation with FR-enriched light (Casal *et al.*, 2000). Thus, to test whether PHYA could specifically limit shade-induced loss of chlorophyll, darkened leaves were illuminated with two different frequencies of FR, either pulsed (pFR) or continuous (cFR), at equal fluences per hour in order to trigger either the VLFR or the HIR, respectively (see Supplementary Figs. S2c and S3a available at *JXB* online). Subjecting darkened leaves to pFR resulted in a similar loss of chlorophyll to that which occurred in the dark treatment; in addition, no difference between Ws wt and *phyA-5* was observed. However, cFR significantly decreased the chlorophyll content in *phyA-5* leaves when compared with Ws wt leaves (Fig. 3a). Intriguingly, the chlorophyll content in Ws wt leaves under cFR did not differ from that in the darkened leaves. Repeating this experiment with another ecotype (Landsberg *erecta* - L*er* wt) and a corresponding *PHYA*-null allele (*phyA-201*) revealed a higher chlorophyll loss in darkness and pFR than under cFR (Fig. 3b). These results differ considerably from those found when adding FR to growth light, which caused about a 25% loss of chlorophyll after 13 d (see Supplementary Figs S3b and S4 available at *JXB* online; Rousseaux *et al.*, 1997; Pons and de Jong-van Berkel, 2004). These experiments confirmed that both darkness and pFR induce a similar reduction in chlorophyll and that shade-induced loss of chlorophyll can be reduced via the *PHYA*-mediated FR-HIR.

### Stability and signalling efficiency of PHYA protein affect the degradation of chlorophyll in shaded leaves

In light, the reduced transcription of *PHYA* and the rapid degradation of PHYA protein (Franklin *et al.*, 2007) lead to a very low abundance of PHYA in mature leaves, thus complicating protein analyses. To avoid this problem and to study how the abundance and characteristics of the PHYA protein in shaded leaves might affect the shade-induced loss of chlorophyll, two additional *phyA* alleles that have been reported to produce PHYA proteins with an altered stability and signalling efficiency were used (Fig. 3c). The first of these alleles, *phyA-401*, has an amino acid substitution of lysine (K) instead of glutamic acid (E) at position 229 (E229K) in the chromophore-binding region (GAF) of PHYA. This mutation causes an increased stability of the PHYA protein in both FR and darkness and enhances the FR-HIR (Dieterle *et al.*, 2005). In agreement with these reports, when shaded, individual L*er phyA-401* leaves retained more chlorophyll than L*er* wt leaves (Fig. 3d). In the second allele, *phyA-302* (E777K; lysine instead of glutamic acid at position 777, in the PAS2 region), the mutation prevents the localization of PHYA into nuclear speckles, resulting in an impaired FR-HIR (Yanovsky *et al.*, 2002). After shading, the chlorophyll loss from L*er phyA-302* leaves was similar to that of the null-mutant L*er phyA-201* (Fig. 3d). Altogether this shows that alterations in PHYA structure and function, as described for the products of the different alleles, correlate with the observed changes in chlorophyll in shaded leaves. It also highlights the need for a proper nuclear localization of the PHYA protein in order for the chlorophyll content to be regulated in response to partial shading of a mature plant.

### *PHYA* modulates the expression of genes related to chlorophyll biosynthesis in shaded leaves

**Fig. 4.**
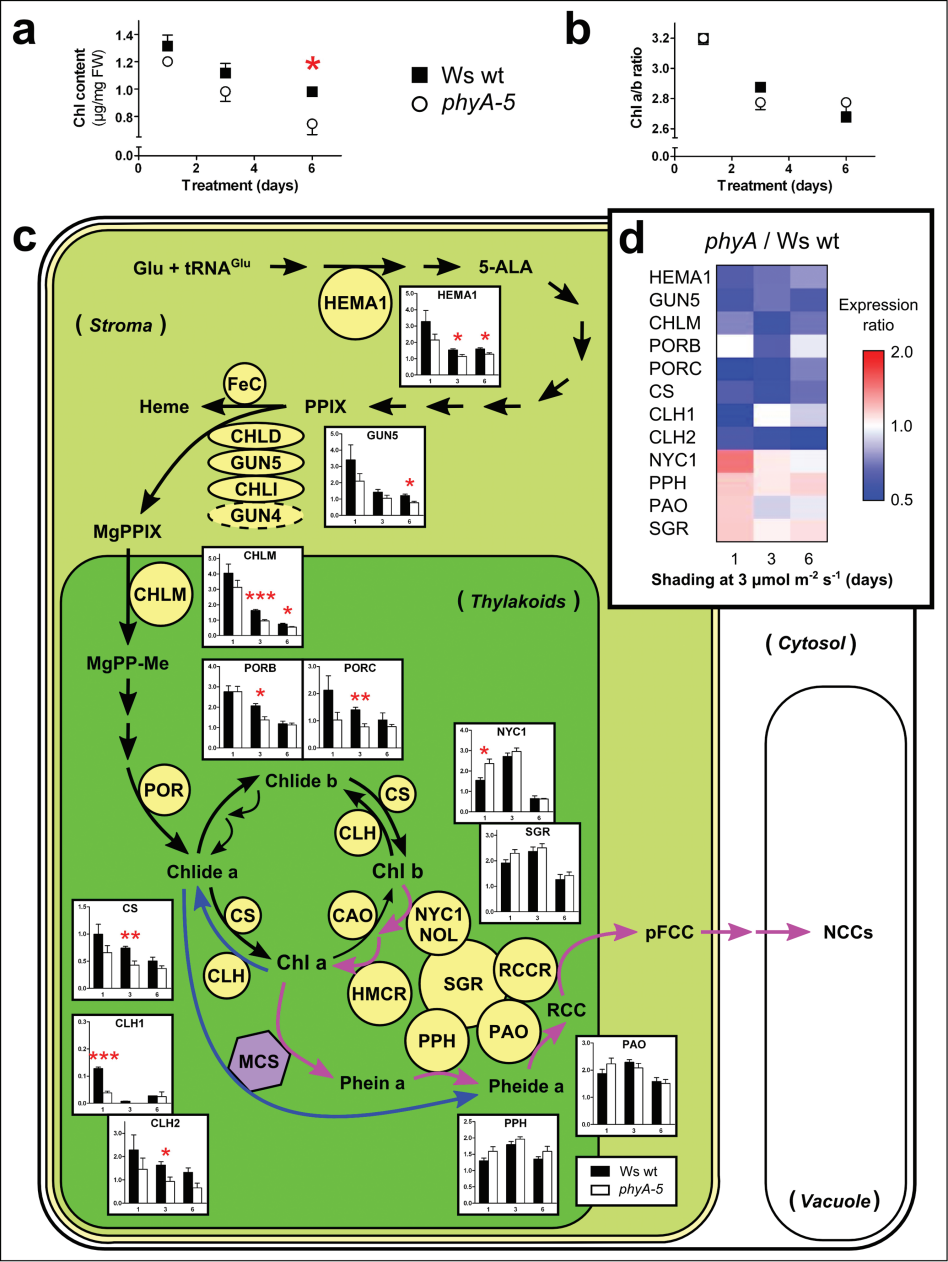
Regulation of the chlorophyll biosynthetic and catabolic pathways in shaded leaves of partially shaded Ws wt and Ws *phyA-5* plants. Changes in (a) chlorophyll content, (b) chlorophyll *a*/*b* ratio, and (c) normalized fold expression of genes involved in the biosynthesis and degradation of chlorophyll in individually shaded leaves (3 μmol m^–2^ s^–1^) for 1, 3 or 6 d. The results in (c) were obtained using qPCR analyses and illustrate the differences in expression between Ws wt (black) and Ws *phyA-5* plants (white). Enlarged figures are available in Supplementary Fig. S6 available at *JXB* online. (d) Heat map representing the transcript abundance expressed as a ratio between *phyA-5* and Ws wt. Values are means ±SEM, *n*=4. Statistically significant *t* tests are notated: *, *P* <0.05; **, *P* <0.01; ***, *P* <0.001.

As mentioned above, the mechanism by which PHYA affects the chlorophyll content in response to partial shade is still unclear. It was therefore hypothesized that the enhanced loss of chlorophyll in shaded *phyA*-mutant leaves was related to changes in either the chlorophyll biosynthetic or catabolic pathways. This was tested by shading Ws wt and *phyA-5* leaves down to 3 μmol m^–2^ s^–1^, an intensity known to generate a significant difference in chlorophyll content after 6 d, while being mild enough to avoid a negative carbon balance and starvation-induced leaf senescence (Fig. 1b) (Brouwer *et al.*, 2012). During the shading treatment, in which leaves were shaded for 1, 3, and 6 d, the chlorophyll content decreased in both genotypes and was significantly lower in the *phyA-5* leaves after 6 d (Fig. 4a). The chlorophyll *a*/*b* ratio also decreased but showed no difference between the two genotypes (Fig. 4b). Furthermore, the expression of genes encoding enzymes associated with either chlorophyll biosynthesis, namely *HEMA1*, *GUN5*, *CHLM*, *PORB*, *PORC*, and *CS* (Beale 1999; Rüdiger, 2002; Tanaka and Tanaka, 2007) or chlorophyll degradation, namely *CLH1*, *CLH2*, *NYC1*, *PPH*, *PAO*, and *SGR* (Schelbert *et al.*, 2009; Sakuraba *et al.*, 2012) were determined by qPCR analyses. The transcript analyses of genes related to chlorophyll biosynthesis revealed that while the expression was reduced in both genotypes, it was significantly lower in the *phyA-5* leaves, particularly after 3 d and 6 d of shade (Fig. 4c). It can be noted here that *PORA* was not included in our study as its expression level in mature leaves was far too low compared with the expression levels of *PORB* and *PORC* (see Supplementary Fig. S5 available at *JXB* online). Meanwhile, the abundance of transcripts from genes related to senescence-associated chlorophyll degradation (*NYC1*, *PPH*, *PAO*, and *SGR*) did not significantly differ between Ws wt and *phyA-5*, except for that of NYC1, which increased only on the first day. Interestingly, the expression of *CLH1* and *CLH2*, which are related to ‘high light’-associated chlorophyll degradation (Bánas *et al.*, 2012), was significantly reduced in *phyA-5* leaves after 1 d and 3 d, respectively. When presenting the relative expression of all the above genes as a ratio between *phyA-5* and Ws wt in a heat map, it became clear that the genes related to chlorophyll biosynthesis and ‘high light’-dependent chlorophyll degradation formed a cluster of down-regulation, whereas the genes related to chlorophyll degradation were only slightly, albeit not significantly, up-regulated (Fig. 4d). Together, these results clearly indicate that the lower chlorophyll content in shaded *phyA-5* leaves originates from an overall down-regulation of the genes encoding enzymes of the chlorophyll biosynthetic pathway and not particularly from an increased expression of genes associated with chlorophyll catabolism.

### In response to partial shading, the lack of PHYA negatively impacts photosynthetic capacity but not dark-respiration

**Fig. 5.**
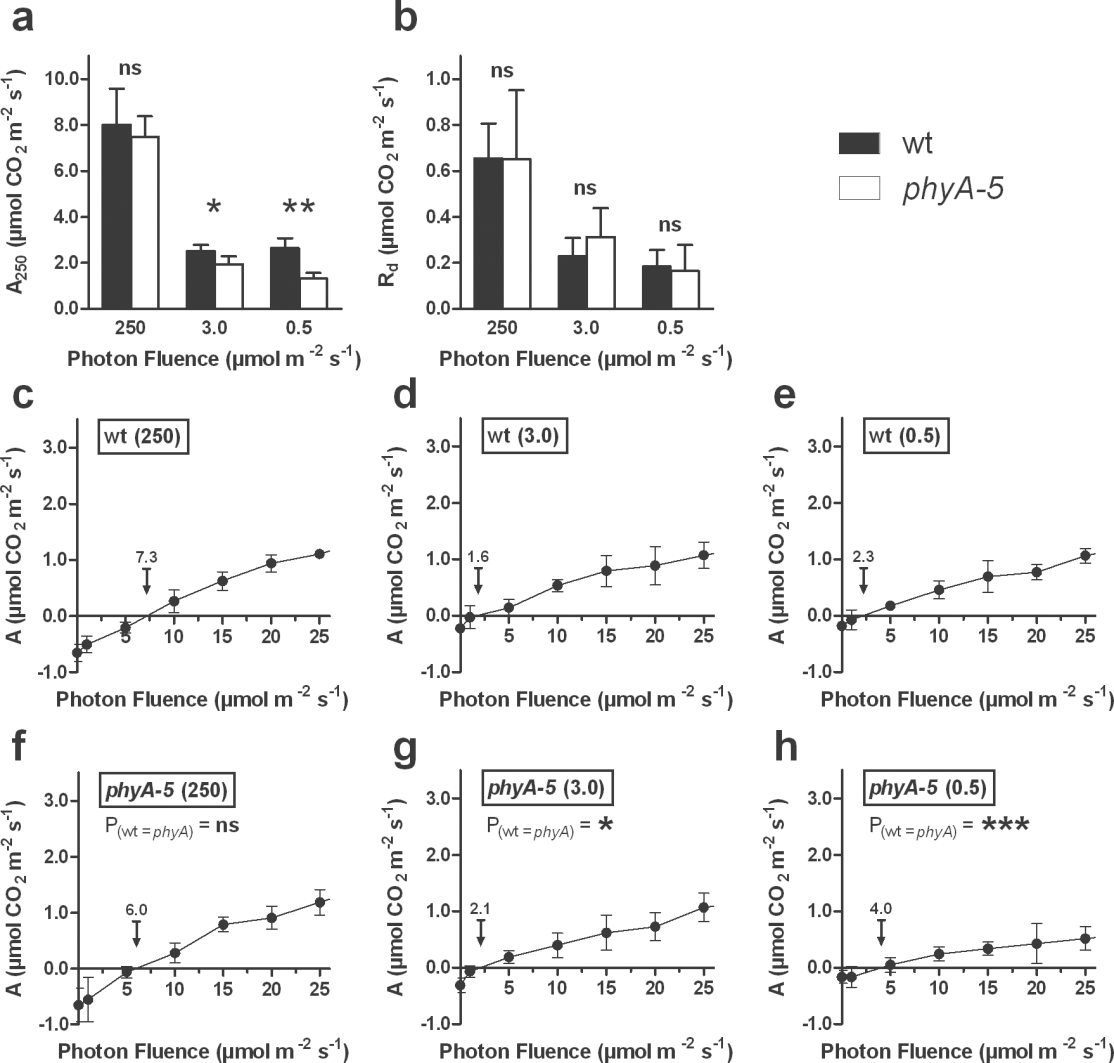
CO_2_-assimilation at (a) growth light intensity (A_250_), (b) dark respiration (R_d_), and (c–h) Light Compensation Point (LCP) in shaded leaves of partially shaded Ws wt and *phyA-5* plants. While the rest of the plant was illuminated at 250 μmol m^–2^ s^–1^ (c, f), individual leaves were shaded to 3.0 μmol m^–2^ s^–1^ (d, g) and to 0.5 μmol m^–2^ s^–1^ (e, h). The arrows indicate the LCP with its value above it. Values are means ±95% CI, *n* ≥5. A_250_ and R_d_ values between wt and *phyA-5* were compared using Mann–Whitney tests, whereas the LCPs between wt and *phyA-5* (c–h) were compared by fitting linear curves between 1 and 20 μmol m^–2^ s^–1^, followed by the application of an Extra sum-of-squares *F* test. Statistically significant differences are notated: *, *P* <0.1; **, *P* <0.01; ***, *P* <0.001; ns, not significant.

It was further questioned whether the altered chlorophyll content observed in the *phyA-5* mutant might have a functional impact on the physiological response of the plant to partial shading. To address this, we determined the photosynthetic activity at growth light (A_250_), the dark respiration (R_d_), and the LCP, the latter being the light intensity value above which the carbon balance between assimilation and respiration is positive. The photosynthetic activity was quantified in leaves that were exposed for 6 d either to growth light or to one of two levels of strong shade (250, 3.0, and 0.5 μmol m^–2^ s^–1^, respectively). Compared with growth light, the photosynthetic capacity of shaded Ws wt leaves was reduced by *c*. 70% (Fig. 5a). Although the shaded leaves from *phyA-5* exhibited a similar trend, the photosynthetic capacity was significantly lower than the one recorded in Ws wt leaves, this being accentuated at the strongest shade treatment (Fig. 5a). Dark respiration decreased by approximately 60% and 70% under 3.0 and 0.5 μmol m^–2^ s^–1^, respectively. However, no differences between the two genotypes were observed (Fig. 5b). Finally, the LCP of Ws wt leaves decreased from approximately 7 μmol m^–2^ s^–1^ in growth light to about 2 μmol m^–2^ s^–1^ in shaded leaves (Fig. 5c–e). However, in *phyA-5* leaves the LCP decreased from approximately 6 μmol m^–2^ s^–1^ in growth light to 2.1 and 4.0 μmol m^–2^ s^–1^, after shading to either 3.0 and 0.5 μmol m^–2^ s^–1^, respectively (Fig. 5f–h). While the LCP of *phyA-5* leaves at growth light was not significantly different from that of Ws wt leaves, the LCPs of shaded *phyA-5* leaves were significantly higher than those of shaded Ws wt leaves. These data show that, under partial shading situations, the lack of *PHYA* negatively affects the photosynthetic capacity of strongly shaded leaves without perturbations of the dark respiration. This, in turn, results in a higher LCP in response to shade.

### Lack of *PHYA* does not significantly enhance the expression of senescence-associated genes

**Fig. 6.**
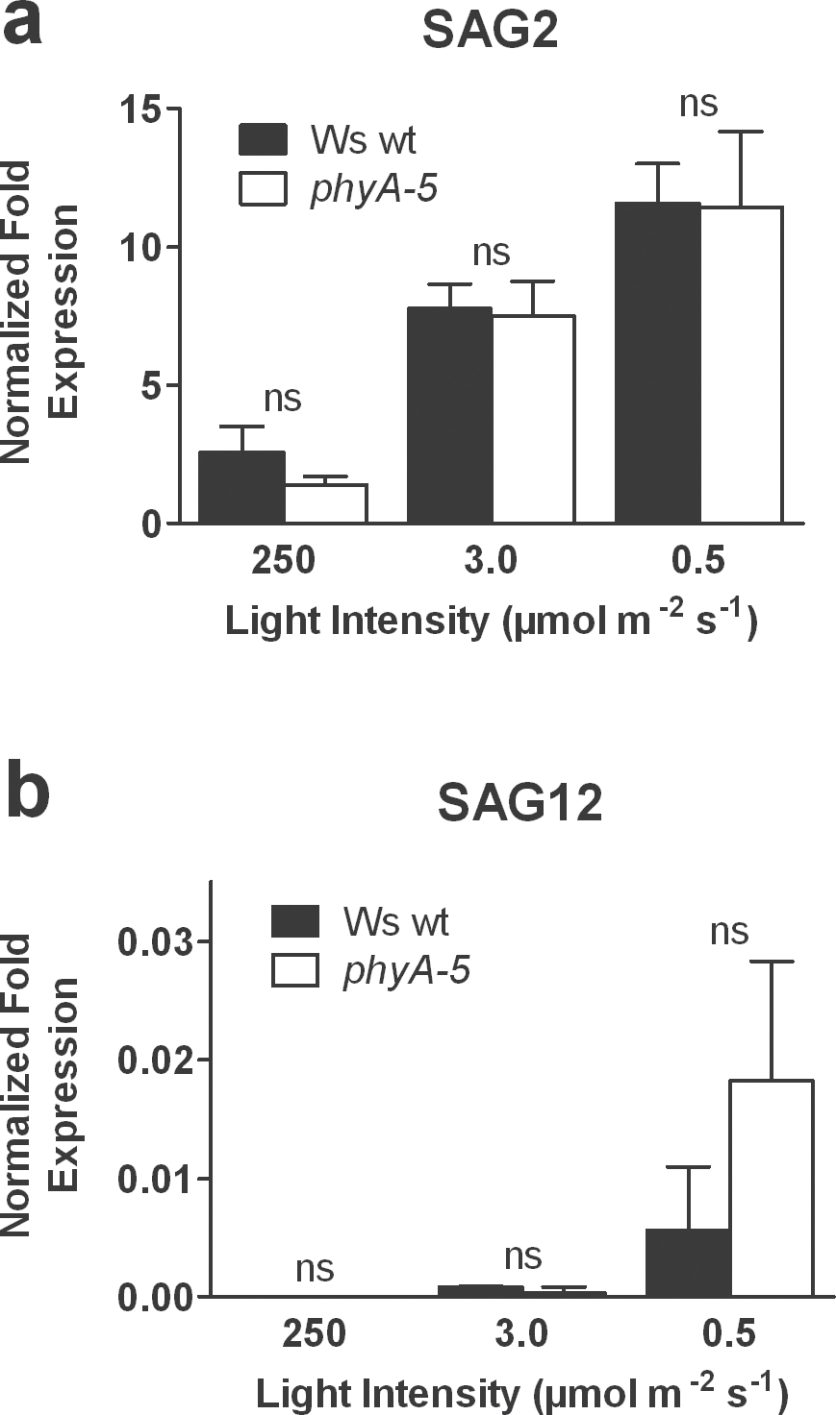
Expression of senescence-associated genes SAG2 (a), and SAG12 (b), in shaded leaves of partially shaded Ws wt and *phyA-5* plants. Leaves were left unshaded (250 μmol m^–2^ s^–1^) or were individually shaded to either 3.0 μmol m^–2^ s^–1^ or 0.5 μmol m^–2^ s^–1^ for 6 d. Values are means ±SEM, *n*=3. ns, non-significant.

In a previous study it was proposed that, in strong shade, leaves can adjust their LCP in order to maintain a positive carbon balance and that would consequently repress the induction of leaf senescence (Brouwer *et al.*, 2012). In the present paper, it has been shown that, in response to strong partial shading, leaves from *phyA-5* mutant plants are compromised in their ability to lower the LCP. Therefore, as a final question it was investigated whether the increased yellowing observed in *phyA-5* plants in response to strong shade correlated with a faster induction of leaf senescence. To this end, qPCR was used to determine the transcript abundance of two additional common molecular markers of leaf senescence: the senescence-associated genes *SAG2* and *SAG12* (Hensel *et al.*, 1993; Lohman *et al.*, 1994). In response to 6 d of shade, the transcript abundance of *SAG2* increased in both Ws wt and *phyA-5*-mutant leaves, 3-fold and 5-fold at 3.0 and 0.5 μmol m^–2^ s^–1^, respectively, when compared with standard growth light conditions (Fig. 6a). By contrast, the transcript abundance of *SAG12* was barely detectable, and after 6 d, only a very weak increase in the transcript abundance in response to 3.0 μmol m^–2^ s^–1^ and 0.5 μmol m^–2^ s^–1^ was noted for both Ws wt and *phyA-5* (Fig. 6b). The fact that, in addition to *PPH*, *PAO*, and *SGR*, none of the two *SAGs* showed any significant difference between Ws wt and *phyA-5* indicates that the absence of *PHYA* does not particularly induce the expression of senescence-associated genes.

## Discussion

Over the past few decades it has been suggested that phytochromes could directly control the induction of leaf senescence (De Greef *et al.*, 1971; Tucker, 1981; Biswal and Biswal, 1984; Rousseaux *et al.*, 1996, 1997; Wingler *et al.*, 2006). However, the signalling and molecular mechanisms by which this might happen have remained unclear. In our present work the aim was to establish a functional connection between phytochrome signalling and the physiological processes underlying the induction of leaf senescence in response to shade. First, and contrary to expectations, it has been shown here that the leaf yellowing processes associated with either complete or partial shading are not connected to the same phytochromes. When plants were completely shaded for 6 d using strong shade (i.e. R: 3.0 μmol m^–2^ s^–1^), *phyB-*mutant plants had a lower leaf chlorophyll content than wt and *phyA* plants (Fig. 2). Conversely, partial shading experiments showed that only *phyA* plants had a lower chlorophyll content in their shaded leaves (Fig. 1). It was previously reported that a leaf could undergo differential metabolic strategies in response to darkness, depending on whether the plant was completely or partially darkened (Keech *et al.*, 2007). When indeed entirely darkened for 6 d, a wt plant had a typical shade-avoidance response that was metabolically characterized by a mild decrease in the photosynthetic capacity and chlorophyll content, and significantly reduced respiration. Conversely, darkening some leaves while leaving the rest of the plant at high irradiance triggered an accelerated senescence in the darkened leaves. This was characterized by an impairment of the photosynthetic machinery coupled with a drastic loss of chlorophyll, while mitochondrial respiration was maintained to support active metabolism during the breakdown of cellular components and the subsequent reallocation of nutrients. The fact that two different phytochromes mediate the chlorophyll content in response to shade, depending on whether the plant is completely or partially shaded, corroborates our previous observations on the differential metabolic strategies in response to darkness. It also strengthens the evidence for the potential involvement of PHYA in mediating the induction of leaf senescence in response to partial shading, but raises a question concerning the extent to which it does so.

### A nuclear PHYA-dependent signalling pathway regulates chlorophyll biosynthesis in response to partial shade

The loss of chlorophyll is recognized as being an important marker for leaf senescence (Ougham *et al.*, 2008) and over the past decade, scientists have gained a better understanding of the mechanisms underlying senescence-associated chlorophyll degradation, notably by identifying the key genes regulating this process (Schelbert *et al.*, 2009; Sakuraba *et al.*, 2012). Therefore, a logical assumption was to associate the enhanced leaf yellowing observed in *phyA*-mutant leaves with the higher rate of chlorophyll degradation that is characteristic of leaf senescence. To our surprise, the faster leaf yellowing observed in partially shaded *phyA*-mutant plants resulted from a reduced expression of genes related to chlorophyll biosynthesis but not to an increased expression of genes related to chlorophyll degradation (Figs 1, 4). These findings argue against the idea of PHYA directly regulating the induction of leaf senescence in response to shade. Direct relationships between gene expression and chlorophyll content may also be nuanced by post-transcriptional and post-translational modifications, particularly for the formation of 5-aminolevulinic acid (ALA) and the branching of the pathway towards chlorophyll and haem (Tanaka and Tanaka, 2011). Nonetheless, it has clearly been shown that the activity of chlorophyll degradation-related PAO is directly proportional to its expression (Pruzinska *et al.*, 2005) and that overall the chlorophyll biosynthesis is mainly regulated at a transcriptional level (Tanaka and Tanaka, 2007; Masuda and Fujita, 2008). In addition, the aforementioned post-translational regulatory mechanisms and assembly of chlorophyll and chlorophyll-binding proteins are suggested only to play a role in facilitating rapid responses, from seconds to minutes, to varying environmental conditions, e.g. sunflecks (Czarnecki and Grimm, 2012; Tanaka and Tanaka, 2011). Therefore, we are confident that the observed modifications of the chlorophyll content after 6 d of shading treatment are, in fact, long-term effects of repressed gene expression rather than the result of fast regulations via post-translational modifications.

Phytochromes are known to translate light signals into an enhanced gene-expression related to the biosynthesis of chlorophyll and photosynthetic protein during de-etiolation (Shin *et al.*, 2009; Franklin and Quail, 2010). In particular, PHYA has been shown to enhance expression of many of these genes under cFR (Tepperman *et al.*, 2001), which agrees with our observations on partial shading under cFR (Fig. 3a, b) and with the fact that PHYA requires FR for signal transduction (Rausenberger *et al.*, 2011). The Pfr spectral form of PHYA promotes the light responses, but *in cellulo* there are two dynamic pools of active phytochrome (Pfr), these being located in the cytosol and in the nucleus respectively, thus suggesting possibilities for both nuclear and cytosolic phytochrome signalling pathways. Paik *et al.* (2012) recently demonstrated that, in seedlings, the cytosolic Pfr form of PHYA and PHYB could interact with a cytosolic phytochrome-binding protein PENTA1 (PNT1) and thereby inhibit the translation of protochlorophyllide reductase A (*PORA*) mRNA. Moreover, the authors also showed that it was only the translation of *PORA* that was regulated by PNT1, and not that of *HEMA1* or *GUN5*. Since our results show that partial plant shading regulates both HEMA1 and GUN5 in a PHYA-dependent manner (Fig. 4c, d), the cytosolic post-transcriptional regulation of chlorophyll biosynthesis genes via PNT1 seems unlikely. Another line of evidence that PHYA regulates chlorophyll levels in response to strong partial shading via a nuclear signalling pathway, comes from the use of *phyA* missense mutants *phyA-401* and *phyA-302*. The *phyA-401* mutant (i.e. *eid4*; Dieterle *et al.*, 2005), which is known to have an increased stability of PHYA as well as a reduced formation of sequestered areas of phytochrome in the cytosol, showed a higher chlorophyll content compared with wt in response to partial shading (Fig. 3d). Using similar reasoning, the *phyA-302* mutant (Yanovski *et al.*, 2002), which exhibits an altered localization of PHYA to nuclear speckles and shows a subsequent impairment of the FR-HIR, phenocopied the null-mutant *phyA-201* in response to partial shading (Fig. 3d). Altogether, this indicates that, when mature *Arabidopsis* plants are subjected to strong partial shading, PHYA, but not PHYB, regulates the expression of the chlorophyll biosynthetic genes via its nuclear localized action in shaded leaves.

The accepted mechanism by which phytochromes transduce their signals through a nuclear localized action is by binding to Phytochrome Interacting Factors (PIFs) and thus targeting them for degradation (Franklin and Quail, 2010; Casal, 2013). Most genes related to the biosynthesis of chlorophyll and photosynthetic protein are induced to similar extents in response to both phytochrome-activating light and the absence of PIF1, PIF3, PIF4, and PIF5 (Shin *et al.*, 2009). Two of these PIFs, PIF1 and PIF3, are known to interact with PHYA and can regulate the expression of genes related to chlorophyll biosynthesis (Huq *et al.*, 2004; Shin *et al.*, 2009; Stephenson *et al.*, 2009; Leivar and Quail, 2011). PIF1 has been shown to act both positively and negatively in the fine-tuning of the chlorophyll biosynthetic pathway (Huq *et al.*, 2004; Moon *et al.*, 2008). In seedlings, PIF1 can stimulate the expression of PORA, PORB, and PORC, which subsequently can bind the free protochlorophyllide and therefore positively regulates the biosynthesis of chlorophyll (Moon *et al.*, 2008). In addition, PIF1 can stimulate the accumulation of haem and thereby inhibit the production of the chlorophyll precursor δ-aminolevulinic acid which, in turn, reduces the production of chlorophyll. PIF3 has also been shown to inhibit chlorophyll biosynthesis specifically, notably by repressing the expression of two key chlorophyll biosynthetic genes, HEMA1 and GUN5 (Shin *et al.*, 2009; Stephenson *et al.*, 2009). Taking together the fact that, in our experiments, the *phyA*-mutant had a lower abundance of *HEMA1*, *GUN5*, and *PORB/PORC* transcripts and that PHYA usually represses the action of PIFs, it is tempting to propose a model in which, in response to partial shading in mature leaves, the Pfr form of PHYA regulates the expression of chlorophyll biosynthetic genes via an interaction with PIF3, but not PIF1. It may yet be found that additional transcription factors are involved in the regulation of the chlorophyll biosynthetic pathway in response to partial plant shading. In that case, further biochemical- and molecular-based studies will be required to determine in detail the exact components involved and how they are orchestrated to achieve such a complex of regulation.

### A lesser capacity to adjust the LCP in response to strong shade is a downstream effect of the lack of *phyA*, but does not promote leaf senescence

When measuring the LCP in shaded leaves of Ws wt and *phyA* plants, a significantly altered ability of *phyA* leaves to lower their LCP in response to strong shading (Fig. 5c–h) was observed. The LCP represents the light intensity below which the carbon assimilated by photosynthesis (Fig. 5a) becomes inferior to the carbon released by respiration (Fig. 5b). Interestingly, there is compelling evidence that the respiratory carbon metabolism is regulated by light (Rasmusson and Escobar, 2007; Igamberdiev *et al.*, 2014) and, recently, *PHYA* has been proposed to regulate mitochondrial respiration by repressing the expression of the A and B subunits of the succinate dehydrogenase (SDH) (Popov *et al.*, 2010). However, even though dark respiration (Rd) decreased drastically in response to partial shading (Fig. 5b), a significant difference in the Rd between wt and *phyA* was not recorded, suggesting a targeted regulation of photosynthesis over respiration by PHYA.

In a previous study, it was proposed that, when strongly shaded, leaves could balance the photochemical efficiencies of their photosystems while minimizing their respiration in order to reduce their LCP and maintain a positive carbon balance (Brouwer *et al.*, 2012). Keeping a positive carbon balance would, in turn, help to repress starvation-induced senescence in the shaded leaf (Buchanan-Wollaston *et al.*, 2005). However, in the present work, the transcript abundance of two specific molecular markers of leaf senescence, *SAG2* and *SAG12*, was not significantly higher in *phyA* than in Ws wt after 6 d of shading treatment (Fig. 6). Despite the faster loss of chlorophyll, this provides further evidence for the argument against a direct control of the induction of leaf senescence by PHYA in response to partial shading.

**Fig. 7.**
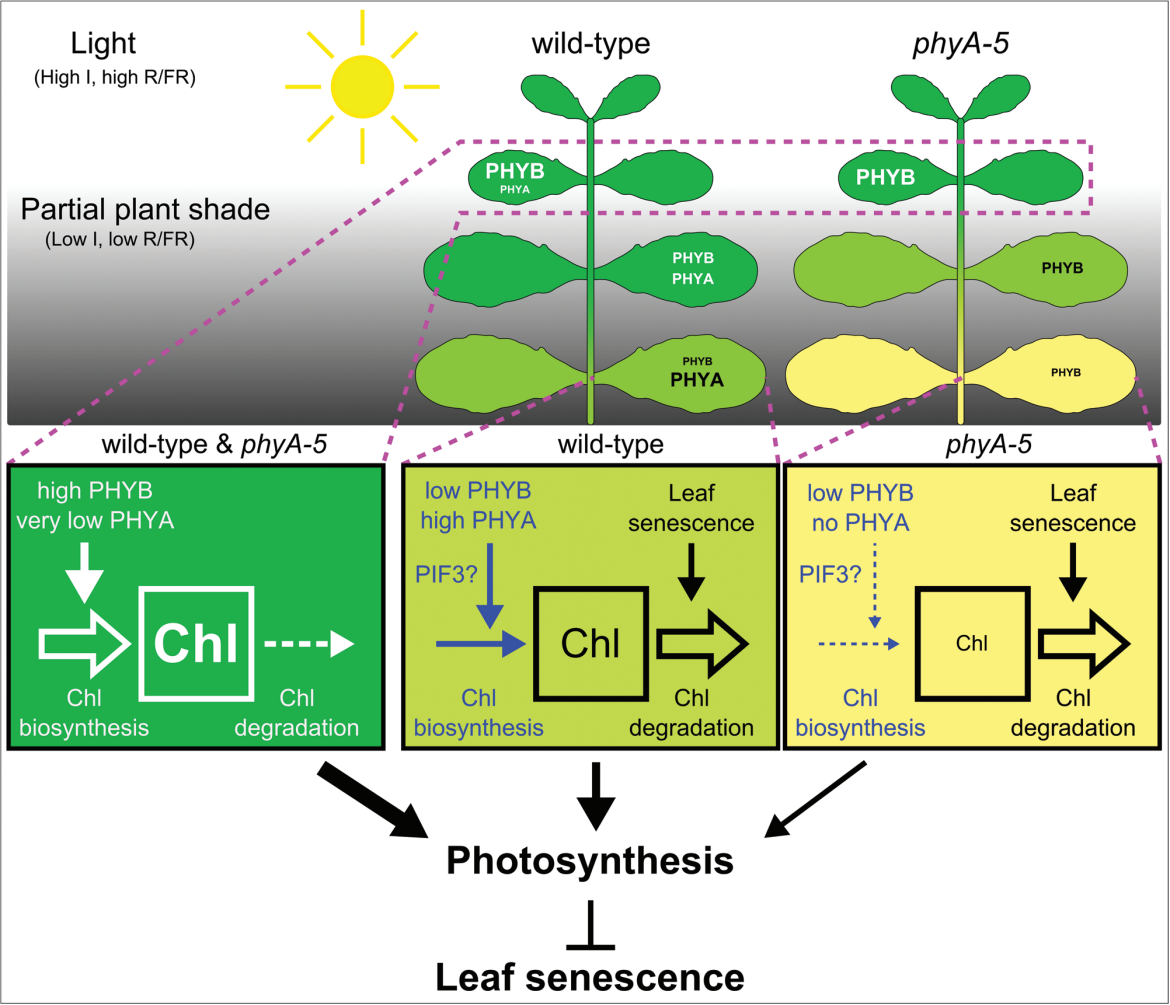
Schematic model representing the impact of phytochrome A on the chlorophyll content of leaves from partially shaded wt and *phyA-5* plants. The accelerated leaf yellowing in *phyA-5* results from an altered regulation between chlorophyll biosynthesis and degradation when compared to wt. In turn, this mis-regulation in the mutant affects the LCP by lowering the photosynthetic capacity. Therefore, it is proposed that PHYA indirectly contributes to the repression of leaf senescence by mediating adjustments to the photosynthetic machinery in order to maintain a positive carbon balance in response to shade. Abbreviations: Chl, chlorophyll; I, light intensity; PHYA, phytochrome A; PHYB, phytochrome B; PIF3, phytochrome interacting factor 3; R/FR, red/far-red ratio.

To conclude, we propose a tentative model (Fig. 7) in which, in response to strong partial shading, the Pfr form of PHYA but not PHYB specifically stimulates the fine-tuning of chlorophyll biosynthesis, probably via its interaction with the bHLH transcription factor PIF3. The physiological impact of this tight regulation of the chlorophyll content enables the shaded leaf to lower its LCP and, consequently, to maintain a positive carbon balance. These mechanisms would therefore prevent the leaf from becoming a sink at a minor energetic cost. By contrast, *phyA*-mutant leaves, being deprived of the capacity to adjust their chlorophyll biosynthesis to a very low irradiance, undergo a faster yellowing that, in turn, negatively impacts the photosynthetic component of their LCP. This could facilitate the induction of senescence in the shaded leaf in the long run. However, additional experiments with a different experimental set-up are needed to assess whether a shading treatment for longer periods of time would be significantly more detrimental for *phyA* than for wt leaves. Nevertheless, our data currently point towards an indirect regulation of the induction of leaf senescence by PHYA in response to partial shading. This work also raises novel questions such as how the overall carbon status of the plant can influence phytochrome-mediated signalling in response to complete or partial shading.

## Supplemental data

Supplementary data are available at *JXB* online.

Supplementary Table S1. Sequences of qPCR-primers.

Supplementary Fig. S1. Chlorophyll content in *phyA phyB* double mutant plants in response to partial plant shading.

Supplementary Fig. S2. Light spectra of the different light conditions.

Supplementary Fig. S3. Experimental set-up used to apply FR (results shown in Fig. 3a, b).

Supplementary Fig. S4. Effect of FR light addition on individual leaves grown under normal light conditions.

Supplementary Fig. S5. Normalized relative expression of *PORA*, *PORB*, and *PORC* during leaf development.

Supplementary Fig. S6. Enlarged figures of the data presented in Fig. 4.

## Supplementary Material

Supplementary Data
