# Supplementary material for: In response to partial plant shading, the lack of phytochrome A does not directly induce leaf senescence but alters the fine-tuning of chlorophyll biosynthesis
Source: J Exp Bot. 2014 Mar 6;65(14):4037–49. doi: 10.1093/jxb/eru060 (PMC4106438; doi:10.1093/jxb/eru060)
Supplement: Supplementary Data [file supp_65_14_4037__index.html]

In response to partial plant shading, the lack of phytochrome A does not directly induce leaf senescence but alters the fine-tuning of chlorophyll biosynthesis — In response to partial plant shading, the lack of phytochrome A does not directly induce leaf senescence but alters the fine-tuning of chlorophyll biosynthesis — Supplementary Data 

# In response to partial plant shading, the lack of phytochrome A does not directly induce leaf senescence but alters the fine-tuning of chlorophyll biosynthesis

## Supplementary Data

Data files

**Files in this Data Supplement:**

- Supplementary Data - Supplementary Data
