# Supplementary material for: In response to partial plant shading, the lack of phytochrome A does not directly induce leaf senescence but alters the fine-tuning of chlorophyll biosynthesis
Source: J Exp Bot. 2014 Mar 6;65(14):4037–49. doi: 10.1093/jxb/eru060 (PMC4106438; doi:10.1093/jxb/eru060)
Supplement: Supplementary Data [file supp_eru060_jexbot114942_file001.pdf]

Supplementary Figures

Table S1. Sequences of qPCR-primers, designed using the indicated gene models. Some of the primers were designed using Quantprime (Arvidsson et al., 2008).

Gene Name	Model	Primer sequence Forward	Reverse	Origin
**Chlorophyll biosynthesis**				
HEMA1	AT1G58290	TCAAGAACTCTGCAGCTGATCG	GCAAGCTTCTCACGCATCTCA	Cheminant *et al.* (2011)
GUN5	AT5G13630.1	AGGCCGAGGATCGATGTAGTTG	GCTCGGTCAAGAAGGTTCATCTGG	own design (Quantprime)
CHLM	AT4G25080	TGGAACCGGTTTGCTCTCGATT	AGCTTCAGCAACCATAGCAGCA	Zhang *et al.* (2012)
PORB	AT4G27440.1	ACTTGCTCAGGTGGTGAGTG	CTCAAAAGAAGCCGAAGCAT	own design (primer3)
PORC	AT1G03630	CACGAGGAAACAGGAGTCACG	CGGAAAAAGAAGCCGAAACA	Cheminant *et al.* (2011)
CS	AT3G51820.1	CTGCTGCTTCAGGGAACTTTC	GGACCAGACATCATCATGCAA	own design (primer3)
**Chlorophyll Degradation**				
CLH1	AT1G19670	CCACATCGCTTCGCATGGTTAC	CACACTTCCAGCATCGTCCAC	Banas *et al.* (2011)
CLH2	AT5G43860	GCATGTCTCTTCTCATGGCTTC	ATCCATTGTGTCTGGTCCGG	Banas *et al.* (2011)
NYC1	AT4G13250.1	GCTATTGTCACTTCCTGGCTAAGG	CTGCATATAACGCCCGTCCTTG	own design (Quantprime)
PPH	AT5G13800.1	TCCATGGGTGAGACCGTTATGG	GCGCATCAGATAGTTCACCACCTC	own design (Quantprime)
PAO	AT3G44880.1	GGCTCGCTCCTTTATCTGAA	TCTCGGGGATTTAACAGCAC	Brouwer *et al.* (2012)
SGR	AT4G22920	GGTGGCCATTTCCTTTTAGA	TCAACAAGTTCCCATCTCCA	Sakuraba *et al.* (2012)
**Senescence-associated**				
SAG2	AT5G60360.3	GGACATTCAGCACAACTGGA	CAAAGGCTTGAGAAGGAAGG	Brouwer *et al.* (2012)
SAG12	AT5G45890.1	AAAGGCGAAGACGCTACTTG	CCTTCATCAGTGCTTGCTCA	Brouwer *et al.* (2012)
**Reference**				
APT1	AT1G27450.2	AGGAAGCCCAAGAAGCTACC	CCTACGTGCATCTCAATCGT	Brouwer *et al.* (2012)
TIP41	AT4G34270	GCTCATCGGTACGCTCTTTT	TCCATCAGTCAGAGGCTTCC	Keech *et al.* (2010)

Figure S1

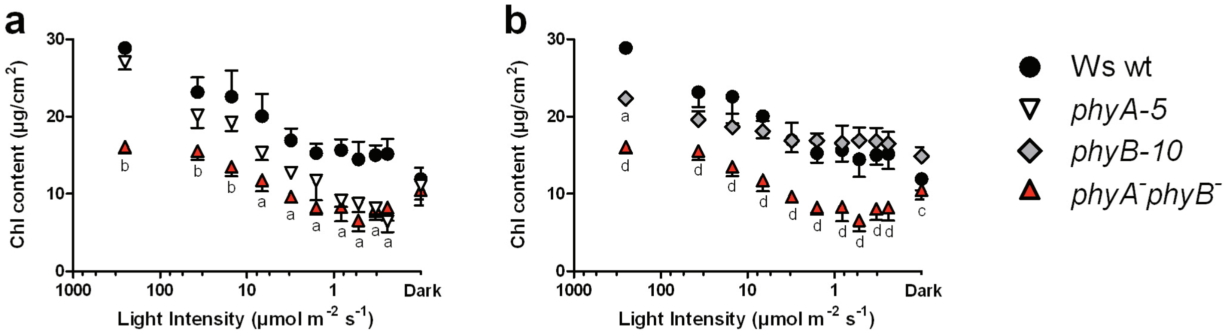

Figure S1. Chlorophyll content in shaded leaves of partially shaded phytochrome mutant plants. Leaves were either uncovered (250 µmol m^{-2} s^{-1}), individually shaded or darkened (Dark) for 6 days. (a) Chlorophyll content in shaded leaves of Ws wt, *phyA-5* and *phyA-5 - phyB-10* plants. (b) Chlorophyll content in shaded leaves of Ws wt, *phyB-10* and *phyA-5 - phyB-10* plants. Values are means ± 95% CI, n ≥ 7. Statistically significant differences ($p < 0.05$) from Kruskall-Wallis with Dunn's multiple comparison tests between treatments are notated: wt 'a'; wt & *phyA-5* 'b'; *phyB-10* 'c'; and wt & *phyB-10* 'd'.

Figure S2

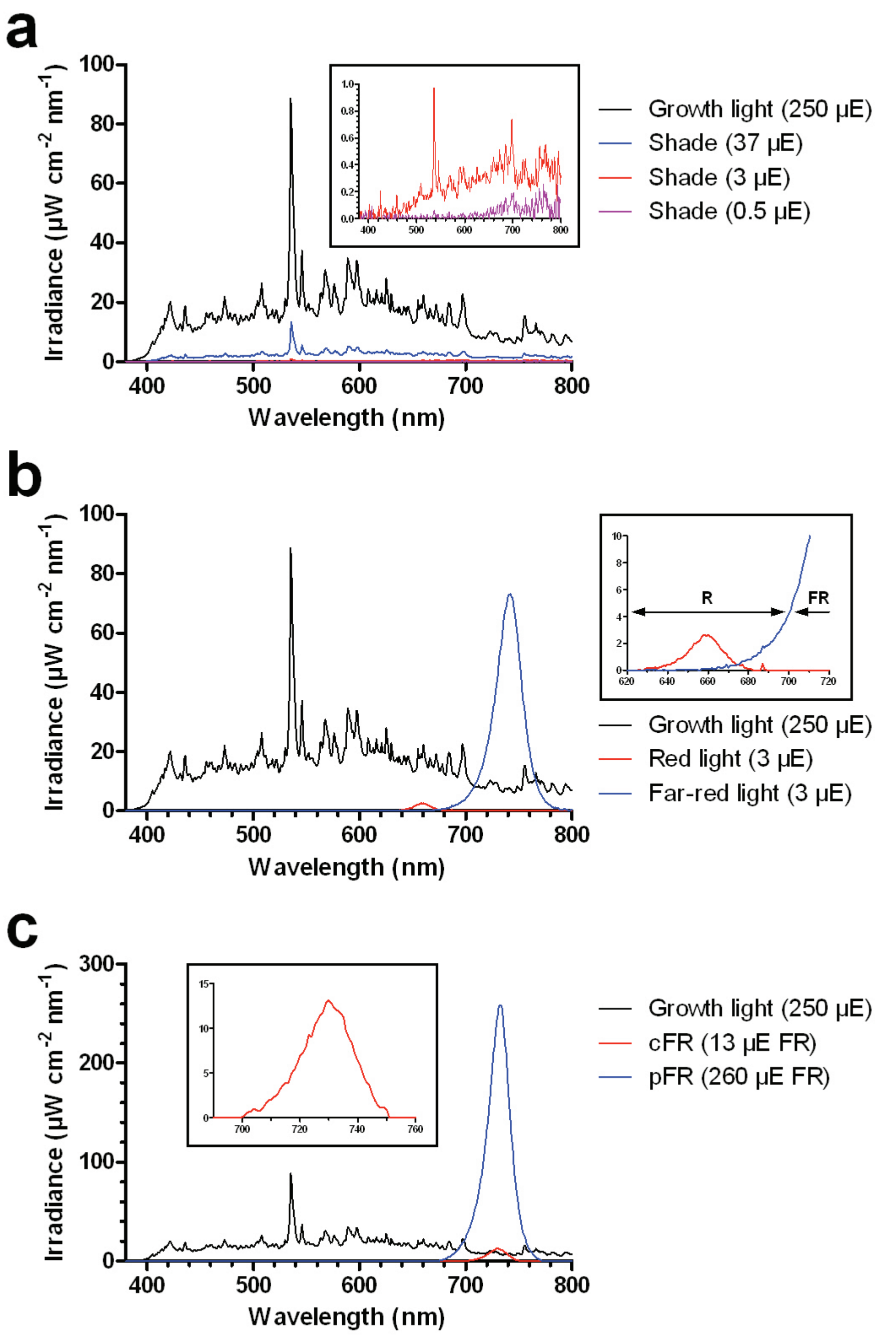

Figure S2. Light spectra of the different light conditions belonging to (a) Growth light (250 µmol m^{-2} s^{-1}) and a selection of light reduction envelopes (37, 3.0 and 0.5 µmol m^{-2} s^{-1}). The insert shows a close-up of the irradiance transmitted by the 3.0 and 0.5 µmol m^{-2} s^{-1} envelopes. (b) Growth light (250 µmol m^{-2} s^{-1}) and growth cabinets for red and far-red light, both at a light intensity of 3 µmol m^{-2} s^{-1}. The insert shows a close-up of the red and far-red light chamber emissions in the 700 nm region, highlighting the emission of the far-red diodes spanning into the visible light, thereby generating a light intensity of 3 µmol m^{-2} s^{-1} and a R/FR ratio of 0.0009 for the far-red chamber. (c) Growth light (250 µmol m^{-2} s^{-1}) and LED-arrays for continuous (13 µmol m^{-2} s^{-1} FR) and pulsed (260 µmol m^{-2} s^{-1} FR) far-red light (pFR and cFR, respectively). The insert shows a close-up of the emission spectrum under the cFR settings in the 690-760 nm region.

Figure S3

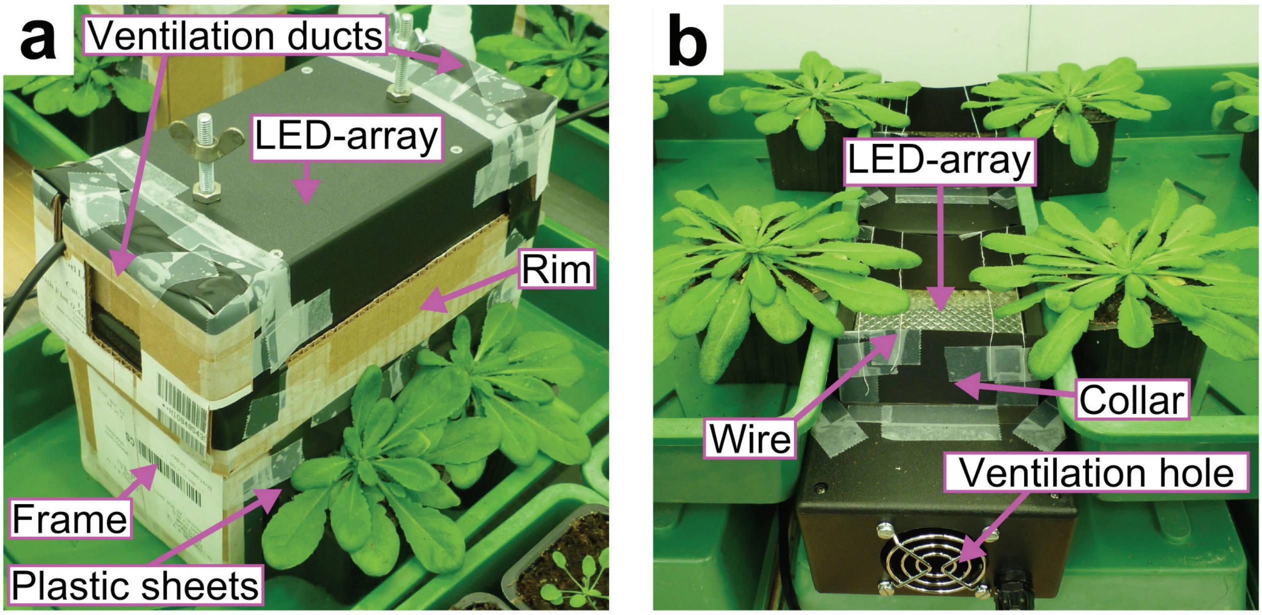

Figure S3. Experimental setups used to determine the effects of FR on leaf chlorophyll content. (a) Setup for partial darkening and regulated application of FR [results shown in Fig. 3a-b]. The frame of the setup is an inverted box that holds a LED-array above the darkened leaves. The top of this frame contains a hole for the LED-array to shine through, while halfway down the frame contains an insert that serves both to avoid light-scatter from the surface below and as placeholder for the potted plants. Between the frame, pots and plants, applied plastic sheets ensure minimal light-scatter from outside to inside and vice versa. The rim around the top, spanning both the box and the LED-array, serves the same purpose as the plastic sheets. Finally, at both ends of the LED-array, ventilation ducts avoid light-scatter through the ventilation holes of the LED-array. (b) Setup for addition of FR to growth light [results shown in Fig. S4]. Far-red light was applied to the abaxial side of the leaf using LED-arrays. Black collars along the edges of the arrays both avoid excessive scatter of the far-red light and serve as frames for the wires that maintain the leaves in a horizontal position over the arrays. Additionally, this figure shows an uncovered ventilation hole, such as mentioned in Fig. S3a.

Figure S4

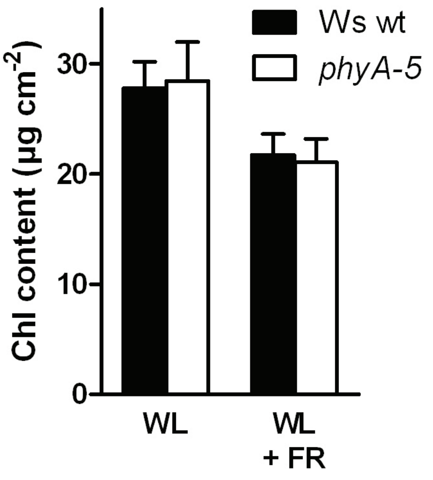

Figure S4. Addition of FR to growth light for 13 days decreases the chlorophyll content in the irradiated leaves and does not depend on the presence of *PHYA*. The additional far-red light (300 µmol m^{-2} s^{-1} FR) was supplied to the abaxial leaf side of a few leaves of 7-week-old plants (Ws wt and *phyA-5*) using upward-facing LED-arrays (MD Electronics, London, UK). The arrays were lined with a 4 cm high, black paper collar to minimize light-scatter and to support overhanging leaves that were further kept in a horizontal position using thin wire (Fig. S3b). The duration of the treatment was 13 days, similar to Rousseaux *et al.*, (1997) and Pons and de Jong van Berkel (2004). Values are means ± 95% CI, n = 6. Statistically significant differences from Mann Whitney tests are notated: *, $p < 0.05$; **, $p < 0.01$.

Figure S5

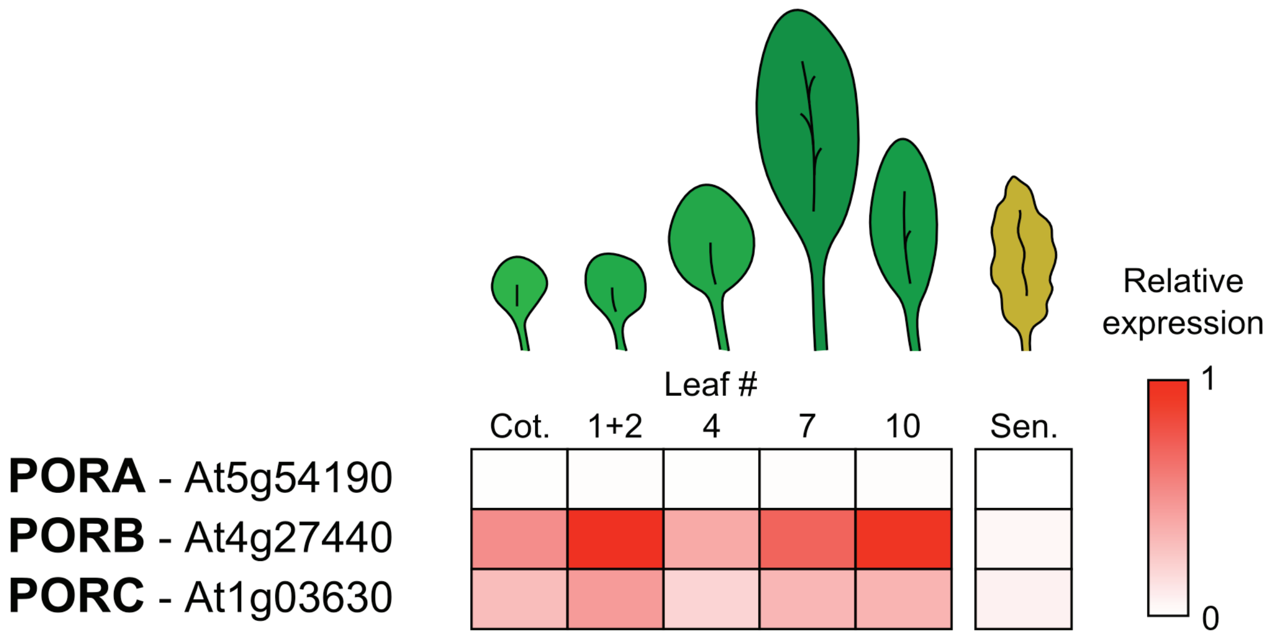

Figure S5. Normalized relative expression of *PORA*, *PORB* and *PORC* during leaf development. Data were collected from eFP-Browser (Winter *et al.*, 2007). All expression values were normalized to the highest expression value of the data set, which was set to 1. Cot: cotyledons, Sen: senescing leaf. Cotyledons and leaves 1+2, 4, 7,10, senescing correspond to the developmental stages described in Schmid *et al.*, 2005. For the leaf at stage 7, the value represents the averaged expression from distal and proximal parts.

Figure S6

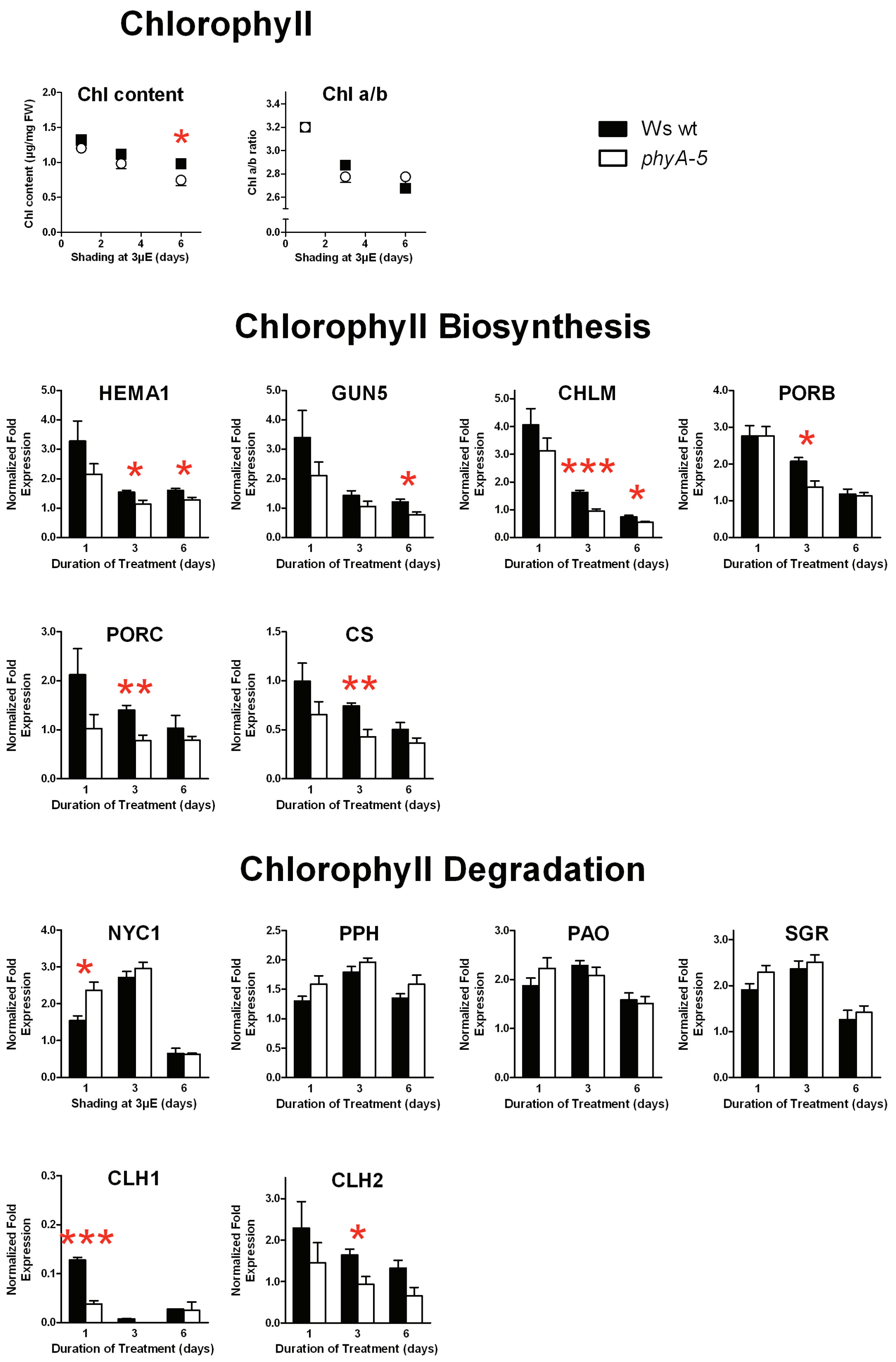

Figure S6. Chlorophyll content, chlorophyll a/b ratio and the normalized fold expression of genes involved in both chlorophyll biosynthesis and chlorophyll degradation that are presented in Fig. 4. Plants of Ws wt and *phyA-5* were partially shaded (3 µmol m^{-2} s^{-1}) for 1, 3 or 6 days before samples were taken for both chlorophyll and qPCR analyses. Values are means ± SEM, n = 4. Statistically significant t-tests are notated: *, $p < 0.05$; **, $p > 0.01$; ***, $p < 0.001$.

Supplementary References

Arvidsson S, Kwasniewski M, Riano-Pachon D, Mueller-Roeber B. 2008. QuantPrime - a flexible tool for reliable high-throughput primer design for quantitative PCR. *BMC Bioinformatics* **9**, 465.

Pons TL, de Jong-van Berkel YEM. 2004. Species-specific variation in the importance of the spectral quality gradient in canopies as a signal for photosynthetic resource partitioning. *Annals of Botany* **94**, 725-732.

Rousseaux MC, Ballare CL, Jordan ET, Vierstra RD. 1997. Directed overexpression of PHYA locally suppresses stem elongation and leaf senescence responses to far-red radiation. *Plant, Cell and Environment* **20**, 1551-1558.

Schmid M, Davison T, Henz S, Pape U, Demar M, Vingron M, Scholkopf B, Weigel D, Lohmann J. 2005. A gene expression map of Arabidopsis thaliana development. *Nature Genetics* **37**, 501 - 506.

Winter D, Vinegar B, Nahal H, Ammar R, Wilson GV, Provart NJ. 2007. An “Electronic Fluorescent Pictograph” Browser for Exploring and Analyzing Large-Scale Biological Data Sets. *PLoS ONE* **2**, e718.
